# Adhesive bonding to dentin in aging and diabetes: a scoping review of substrate-related changes and bonding outcomes

**DOI:** 10.1186/s12903-026-09208-3

**Published:** 2026-07-11

**Authors:** Akshatha Chatra, Arun Mayya, Elstin Anbu Raj S, Kishore Ginjupalli, Shashi Rashmi Acharya

**Affiliations:** 1 Department of Conservative Dentistry and Endodontics, Manipal College of Dental Sciences, Manipal Academy of Higher Education, Manipal, Karnataka 576104 India; 2 Department of Health Technology and Informatics, Prasanna School of Public Health, Manipal Academy of Higher Education, Manipal, Karnataka 576104 India; 3 Department of Dental Materials, Manipal College of Dental Sciences, Manipal Academy of Higher Education, Manipal, Karnataka 576104 India

**Keywords:** Adhesive dentistry, Aging, Dentin bonding, Diabetes mellitus

## Abstract

**Background:**

Aging and diabetes mellitus are associated with structural and biochemical changes in dentin, including altered collagen organization, dentinal sclerosis, tubule occlusion, mineral changes, and, in some contexts, increased non-enzymatic collagen glycation. These substrate changes may influence the performance and durability of adhesive restorative materials. However, the extent to which bonding outcomes can be directly attributed to advanced glycation end products (AGEs) remains uncertain, as AGE levels are rarely measured in adhesive studies.

**Methods:**

This scoping review followed the Joanna Briggs Institute methodology and was reported in accordance with the PRISMA-ScR guidelines. PubMed, Scopus, Web of Science, and Embase databases were searched from inception to December 1, 2025. Eligible studies evaluated bond strength, interfacial morphology, microleakage, mineral content, or remineralization in dentin affected by aging, diabetes, or experimental glycation. Study selection and data charting were independently conducted by two reviewers.

**Results:**

Of the 2615 records identified, 16 primary experimental studies met the inclusion criteria, most of which were in vitro investigations using extracted human teeth. Fifteen studies reported bond strength outcomes, and two assessed microleakage. Only four of the included studies directly evaluated diabetic dentin, and these studies generally reported reduced bond strength, particularly in type 1 diabetes. Most of the included studies evaluated age-related dentin, and the findings were heterogeneous, with many contemporary adhesives showing minimal differences between young and aged dentin. None of the included studies directly quantified AGE levels in dentin before adhesive testing.

**Conclusion:**

Dentin alterations associated with aging and diabetes may influence adhesive performance; however, the available evidence is heterogeneous and does not allow these effects to be directly attributed to AGE accumulation. Therefore, aging and diabetes should be interpreted as distinct dentin-modifying contexts rather than equivalent AGE-mediated substrates. Further studies incorporating biochemical assessment of dentin, participant-level clinical variables, and standardized durability testing are needed.

**Scoping review registration:**

The protocol for this scoping review was registered on the Open Science Framework (OSF) on 16th January 2026 (https://doi.org/10.17605/OSF.IO/JWA6H).

**Supplementary Information:**

The online version contains supplementary material available at https://doi.org/10.1186/s12903-026-09208-3.

## Introduction

Adhesive dentistry aims to create a durable resin-dentin interface by infiltrating a demineralized collagen matrix with resin monomers; the evolution from multistep etch-and-rinse systems to simplified universal adhesives has streamlined clinical procedures but necessitates evaluation of their performance on dentin [1]. However, inadequate resin infiltration leaves collagen fibrils unprotected, making the hybrid layer susceptible to hydrolysis and enzymatic breakdown and undermining the longevity of composite restorations [2]. Acid etching and self-etch adhesives activate endogenous matrix metalloproteinases (MMPs) that degrade collagen fibrils within the hybrid layer; chlorhexidine pretreatment can inhibit these enzymes and improve bond durability [3].

In diabetes mellitus, hyperglycemia impairs mineralized matrix formation, reduces alkaline phosphatase activity and accelerates the non-enzymatic glycation of dentinal proteins, leading to the accumulation of advanced glycation end products (AGEs) that hinder osteoblast differentiation and mineralization [4]. AGEs trigger low-grade chronic inflammation and are associated with periodontal and dental pathologies, and salivary AGE levels are correlated with systemic and oral disease burden [5]. In dentin, AGEs cross-link collagen via Maillard reactions; accumulation of pentosidine and other AGE cross-links increases hardness but makes dentin more brittle, and levels rise with age and hyperglycemia [6]. An age- estimation study demonstrated that the pentosidine concentration in dentine strongly correlates with chronological age and is greater in diabetic individuals [7]. Advanced glycation and mineral deposition in aged dentin occlude the dentinal tubules, increase the modulus and hardness, and reduce the flexural strength and toughness [8]. Non-enzymatic glycation of dentinal collagen introduces cross-links that alter mechanical properties and increase susceptibility to caries [9]. Comprehensive analyses of aged hard tissues revealed that AGE cross-links accumulate in intertubular collagen and dentinal tubules, causing fragility, whereas mineral deposition narrows tubule lumens and decreases porosity, increasing stiffness but decreasing fracture toughness [10]. Natural aging also reduces dentinal permeability through tubule mineralization, influencing adhesive performance [11].

Importantly, aging and diabetes should not be regarded as biologically equivalent dentin-modifying conditions. Aging is a physiological process characterized by gradual dentinal sclerosis, reduced tubular permeability, mineral deposition, and cumulative collagen changes. In contrast, diabetes mellitus is a systemic metabolic disease associated with chronic hyperglycemia, oxidative stress, inflammatory dysregulation, altered mineral metabolism, and variable disease duration and glycemic control. Although AGE accumulation may occur in both contexts, the pathways by which aging and diabetes affect dentin structure and adhesive bonding are not identical. Therefore, in this review, AGEs are considered a plausible mechanistic contributor rather than a directly demonstrated cause of altered bonding outcomes. The findings from aged dentin, diabetic dentin, and experimentally glycated dentin are interpreted as related but distinct evidence streams.

These substrate-related changes adversely affect resin-dentin interactions and hybrid layer formation and can compromise the effectiveness of adhesive dentistry. Compared with healthy teeth, diabetic teeth exhibit significantly lower push-out bond strength to root canal sealers and restoratives [12]. Compared with conventional resin composites, self-adhesive resin composites have inferior bond strengths, microleakage scores and marginal adaptations; however, preconditioning with universal adhesives improves outcomes [13]. An ex vivo study revealed that shear bond strength values were lower in diabetic dentin than in non-diabetic dentin and that the removal of bioceramic sealer residues with dry cotton was insufficient; ultrasonic cleaning improved the bond strength in both groups [14]. Another experimental study on fiber posts reported that dentin type, resin cement and irrigation solution significantly influenced push-out bond strength; chlorhexidine irrigation increased bond strength, whereas saline alone yielded the lowest values in diabetic dentin [15]. Collectively, these findings suggest that substrate changes associated with diabetes and aging may negatively affect adhesion; however, the specific contribution of AGE accumulation cannot be confirmed unless AGE levels are directly quantified.

Given the increasing prevalence of diabetes and aging populations worldwide, clinicians will increasingly encounter dentin substrates affected by age-related sclerosis, altered permeability, metabolic disease, and potentially increased collagen glycation. Despite a growing body of research on bond strength, interfacial morphology, microleakage, and remineralization in aged and diabetic dentin, the literature remains fragmented, and the role of AGE accumulation remains insufficiently characterized. Therefore, this scoping review aims to systematically map the extent and nature of evidence on adhesive performance in dentin affected by aging, diabetes, or experimental glycation across in vitro, ex vivo, and clinical contexts, including bond strength, interfacial morphology, microleakage, mineral content, and remineralization. A secondary aim was to identify whether included studies directly assessed AGE accumulation or other biochemical markers that could explain observed bonding outcomes.

## Materials and methods

### Protocol and research question

This scoping review followed the methodological frameworks described by Arksey and O’Malley and subsequent extensions developed by the Joanna Briggs Institute (JBI) [16, 17]. The JBI and PRISMA-ScR guidelines were consulted to guide protocol development [17]. The protocol for this scoping review was registered on the Open Science Framework (OSF) on 16th January 2026 [18]. The protocol for this review was designed around the Population-Concept-Context (PCC) framework. The research question was “What is known about the bonding performance of dental adhesives on dentin substrates affected by aging, diabetes, or experimental glycation, and to what extent have these studies directly assessed AGE accumulation or related biochemical substrate changes?” The objectives were to map the extent and nature of evidence on bond strength, interfacial morphology, microleakage, mineral content, and remineralization of resin- and ionomer-based adhesive systems when used on dentin affected by aging, diabetes, or experimental glycation, while distinguishing these substrate categories during data charting and synthesis.

### Eligibility criteria

Eligibility criteria were defined a priori via the PCC framework. We included studies evaluating dentin from diabetic patients, aged or geriatric donors, or experimental models in which dentin glycation was induced chemically or otherwise. These categories were considered separately because aging, diabetes, and experimental glycation represent distinct biological contexts, even though AGE accumulation may be relevant to more than one of them. Eligible concepts included adhesive performance on modified dentin substrates, including bond strength, interfacial morphology, microleakage, mineral content, and remineralization. Laboratory-based experiments, pre-clinical animal studies and clinical or ex vivo studies using extracted human teeth were eligible. Studies were excluded if they analyzed enamel or other tissues without dentin evaluation, examined systemic AGE effects without assessing dentin, or assessed dentin without explicitly relating findings to diabetes, aging, or AGE induction. Systematic reviews, conference abstracts, editorials and other secondary sources were excluded.

### Information sources and search strategy

The search strategy was developed in consultation with an experienced librarian. The databases searched included PubMed, Scopus, Web of Science and Embase from database inception to December 1, 2025, for combinations of controlled vocabulary (MeSH/Index terms) and free‑text keywords. The PubMed strategy used terms for dentin (e.g., Dentin, dentin, dentine) combined with a broad set of terms for advanced glycation end products and related markers (e.g., “Glycation End Products, Advanced”, “advanced glycation end product*”, AGE‑modified, glycat, “Maillard Reaction”, pentosidine, carboxymethyllysine, carboxyethyllysine, CML, CEL, methylglyoxal, glyoxal, ribose) and terms for diabetes or aging (e.g., “Diabetes Mellitus”, diabet, Aged, “older adult*”, geriatric). These were combined using Boolean operators with dentin adhesive outcomes, including “Dental Bonding”, bond strength, microtensile, micro‑tensile, µTBS/uTBS, resin‑dentin interface, hybrid layer, interfacial morphology, “Dental Leakage”, microleakage, nanoleakage, “Tooth Remineralization”, remineralization, mineral content and mineral density. No language or date restrictions were applied. The reference lists of the included articles were manually screened to identify additional studies. Grey literature sources were not searched because this review focused on full-text, peer-reviewed primary experimental studies with sufficient methodological and outcome details to allow extraction of dentin substrate characteristics, adhesive protocols, and bond-strength or interfacial outcomes. Conference abstracts and other sources without complete methodological reporting were excluded because they were unlikely to provide the level of detail required for data charting. The search was performed iteratively and modified as necessary in accordance with JBI recommendations [19]. The specific search strategies for each database are summarized in Table 1.

**Table 1:** Search strategy and number of records retrieved from different databases

Database	Search strategy	Identified articles
PubMed	(Dentin[Mesh] OR dentin*[tiab] OR dentine[tiab] ) AND (“Glycation End Products, Advanced“[Mesh] OR “advanced glycation end product*“[tiab] OR AGE-modified[tiab] OR glycat*[tiab] OR “Maillard Reaction“[Mesh] OR Maillard[tiab] OR pentosidine[tiab] OR carboxymethyllysine[tiab] OR carboxyethyllysine[tiab] OR CML[tiab] OR CEL[tiab] OR methylglyoxal[tiab] OR glyoxal[tiab] OR ribose[tiab] OR (Diabetes Mellitus[Mesh] OR diabet*[tiab] OR Aged[Mesh] OR elder*[tiab] OR “older adult*“[tiab] OR geriatric*[tiab])) AND (“Dental Bonding“[Mesh] OR “bond strength“[tiab] OR microtensile*[tiab] OR “micro-tensile“[tiab] OR µTBS[tiab] OR uTBS[tiab] OR “resin-dentin interface“[tiab] OR “hybrid layer“[tiab] OR “interfacial morpholog*“[tiab] OR “Dental Leakage“[Mesh] OR microleakage[tiab] OR nanoleakage[tiab] OR “Tooth Remineralization“[Mesh] OR remineraliz*[tiab] OR “mineral content“[tiab] OR “mineral densit*“[tiab])	194
Scopus	(((INDEXTERMS(dentin) OR TITLE-ABS(dentin*) OR TITLE-ABS(dentine))) AND (((INDEXTERMS(“glycation end products, advanced”) OR TITLE-ABS(“advanced glycation end product*”) OR TITLE-ABS(AGE-modified) OR TITLE-ABS(glycat*) OR INDEXTERMS(“Maillard Reaction”) OR TITLE-ABS(Maillard) OR TITLE-ABS(pentosidine) OR TITLE-ABS(carboxymethyllysine) OR TITLE-ABS(carboxyethyllysine) OR TITLE-ABS(CML) OR TITLE-ABS(CEL) OR TITLE-ABS(methylglyoxal) OR TITLE-ABS(glyoxal) OR TITLE-ABS(ribose))) OR ((INDEXTERMS(“diabetes mellitus”) OR TITLE-ABS(diabet*) OR INDEXTERMS(aged) OR TITLE-ABS(elder*) OR TITLE-ABS(“older adult*”) OR TITLE-ABS(geriatric*))))) AND ((INDEXTERMS(“Dental Bonding”) OR TITLE-ABS(“bond strength”) OR TITLE-ABS(microtensile*) OR TITLE-ABS(micro-tensile) OR TITLE-ABS(TBS) OR TITLE-ABS(uTBS) OR TITLE-ABS(“resin-dentin interface”) OR TITLE-ABS(“hybrid layer”) OR TITLE-ABS(“interfacial morpholog*”) OR INDEXTERMS(“Dental Leakage”) OR TITLE-ABS(microleakage) OR TITLE-ABS(nanoleakage) OR INDEXTERMS(“Tooth Remineralization”) OR TITLE-ABS(remineraliz*) OR TITLE-ABS(“mineral content”) OR TITLE-ABS(“mineral densit*”)))	397
Web of Science	(((ALL=dentin OR (TI=dentin* OR AB=dentin*) OR (TI=dentine OR AB=dentine))) AND (((ALL="glycation end products, advanced” OR (TI="advanced glycation end product*” OR AB="advanced glycation end product*”) OR (TI = AGE-modified OR AB = AGE-modified) OR (TI=glycat* OR AB=glycat*) OR ALL="Maillard Reaction” OR (TI=Maillard OR AB=Maillard) OR (TI=pentosidine OR AB=pentosidine) OR (TI=carboxymethyllysine OR AB=carboxymethyllysine) OR (TI=carboxymethyllysine OR AB=carboxymethyllysine) OR (TI = CML OR AB = CML) OR (TI = CEL OR AB = CEL) OR (TI=methylglyoxal OR AB=methylglyoxal) OR (TI=glyoxal OR AB=glyoxal) OR (TI=ribose OR AB=ribose))) OR ((ALL="diabetes mellitus” OR (TI=diabet* OR AB=diabet*) OR ALL=aged OR (TI=elder* OR AB=elder*) OR (TI="older adult*” OR AB="older adult*”) OR (TI=geriatric* OR AB=geriatric*))))) AND ((ALL="Dental Bonding” OR (TI="bond strength” OR AB="bond strength”) OR (TI=microtensile* OR AB=microtensile*) OR (TI=micro-tensile OR AB=micro-tensile) OR (TI = TBS OR AB = TBS) OR (TI=utis OR AB=utis) OR (TI="resin-dentin interface” OR AB="resin-dentin interface”) OR (TI="hybrid layer” OR AB="hybrid layer”) OR (TI="interfacial morpholog*” OR AB="interfacial morpholog*”) OR ALL="Dental Leakage” OR (TI=microleakage OR AB=microleakage) OR (TI=nanoleakage OR AB=nanoleakage) OR ALL="Tooth Remineralization” OR (TI=remineraliz* OR AB=remineraliz*) OR (TI="mineral content” OR AB="mineral content”) OR (TI="mineral densit*” OR AB="mineral densit*”)))	938
Embase	(‘dentin’/exp OR ’dentin*’:ti, ab, kw OR ’dentine’:ti, ab, kw) AND (‘advanced glycation end product’/exp OR ’advanced glycation end product*’:ti, ab, kw OR ’age-modified’:ti, ab, kw OR ’glycat*’:ti, ab, kw OR ’glycation’/exp OR ’maillard’:ti, ab, kw OR ’pentosidine’:ti, ab, kw OR ’carboxymethyllysine’:ti, ab, kw OR ’carboxyethyllysine’:ti, ab, kw OR ’cml’:ti, ab, kw OR ’cel’:ti, ab, kw OR ’methylglyoxal’:ti, ab, kw OR ’glyoxal’:ti, ab, kw OR ’ribose’:ti, ab, kw OR ’diabetes mellitus’/exp OR ’diabet*’:ti, ab, kw OR ’aged’/exp OR ’elder*’:ti, ab, kw OR ’older adult*’:ti, ab, kw OR ’geriatric*’:ti, ab, kw) AND (‘dental bonding’/exp OR ’bond strength’:ti, ab, kw OR ’microtensile*’:ti, ab, kw OR ’micro-tensile’:ti, ab, kw OR ’tbs’:ti, ab, kw OR ’utbs’:ti, ab, kw OR ’resin-dentin interface’:ti, ab, kw OR ’hybrid layer’:ti, ab, kw OR ’interfacial morpholog*’:ti, ab, kw OR ’tooth disease’/exp OR ’microleakage’:ti, ab, kw OR ’nanoleakage’:ti, ab, kw OR ’dental procedure’/exp OR ’remineraliz*’:ti, ab, kw OR ’mineral content’:ti, ab, kw OR ’mineral densit*’:ti, ab, kw)	1086

### Study selection

All records identified through electronic searches were imported into Rayyan (Rayyan Systems Inc.) [20], an open-access web-based review management tool, where automatic and manual duplicate removal was performed. Titles and abstracts were screened independently by two reviewers (A.C. and A.M.) against the inclusion criteria. When eligibility was unclear, the full texts were retrieved. Full-text screening was conducted independently by the same reviewers, and disagreements were resolved by consensus or consultation with a third reviewer [19].

### Data charting and extraction

The data were charted via a piloted and iteratively refined extraction form. Two reviewers independently extracted all the relevant information and compared the entries to resolve discrepancies through discussion. The following variables were extracted from each study: bibliographic details (author, year, country), study characteristics (design, setting), dentin type (human diabetic, geriatric, in vitro AGE-induced, or animal models), method of AGE induction (chemical, biological, or naturally occurring), adhesive system evaluated (etch-and-rinse, self-etch, universal, or ionomeric materials), sample characteristics, testing methodology, and outcome measures, including bond strength results, interfacial morphology, microleakage performance, mineral content or remineralization findings, and authors’ conclusions. Where available, participant- and tooth-level variables that could influence dentin quality were also charted, including chronological age, diabetes type, duration of diabetes, glycemic control or HbA1c status, presence of other systemic diseases, medication use, smoking status, tooth type, dentin location, degree of sclerosis, and storage conditions. When these variables were not reported, they were recorded as ‘not reported’ and considered during interpretation of the evidence. Additional fields included key methodological notes and reviewer comments. The authors of the included studies were not contacted for additional data. A detailed overview of the experimental protocols and extracted variables for all included studies is provided in Supplementary Table 1.

### Data synthesis

The extracted data were synthesized descriptively. The studies are summarized in tabular form to present their bibliographic characteristics, study designs, dentin sources, methods of AGE induction, adhesive systems tested, sample characteristics, and outcome measures. A numerical summary was generated to illustrate the number of studies addressing each outcome category, including bond strength, interfacial morphology, microleakage, and mineral content or remineralization. This was followed by a narrative synthesis describing the observed trends in adhesive behavior in dentin affected by aging, diabetes, or experimental glycation. No critical appraisal was undertaken to map evidence (JBI scoping guidance).

### Reporting

**Fig. 1:**
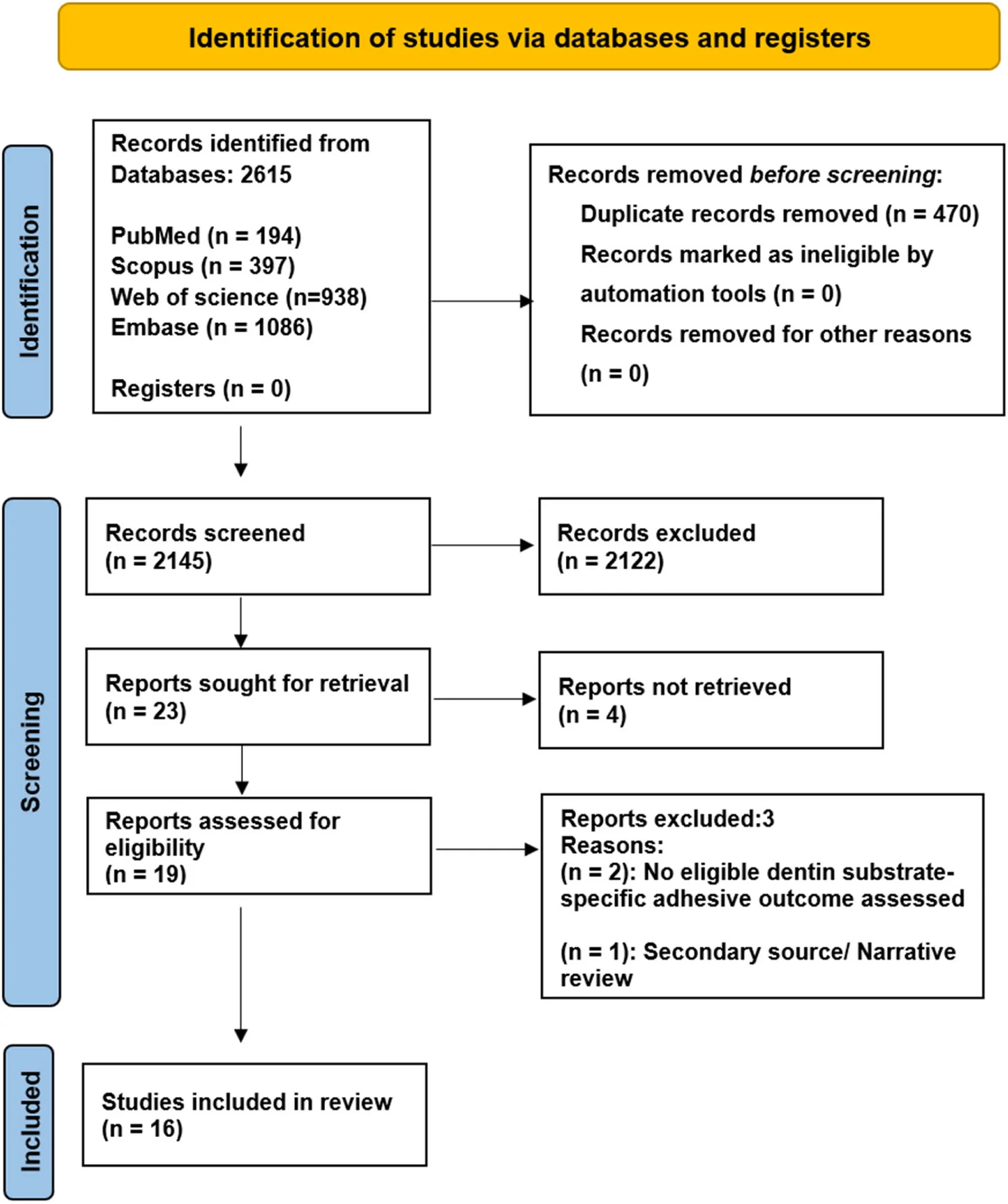
PRISMA 2020 flow diagram illustrating the study selection process for this scoping review

This review is reported in accordance with the Preferred Reporting Items for Systematic Reviews and Meta-Analyses Extension for Scoping Reviews (PRISMA-ScR) checklist [16]. The PRISMA-ScR checklist (Supplementary Table 2) and the PRISMA flow diagram (Fig. 1) accompany the manuscript.

## Results

### Study selection and characteristics

The electronic search across PubMed, Scopus, Web of Science, and Embase yielded 2615 records (PubMed = 194, Scopus = 397, Web of Science = 938 and Embase = 1086). After the removal of 470 duplicates, 2145 titles and abstracts were screened. A total of 2122 records were excluded. 23 full-text articles were sought for retrieval, of which 4 were not retrieved. 19 reports were assessed for eligibility, of which 16 primary experimental studies met the inclusion criteria and were included in the final synthesis. The PRISMA flowchart in Fig. 1 illustrates the entire process. All 16 included studies were experimental investigations. Fifteen studies used extracted human teeth only [4, 11, 15, 21–32], and one study used both human and bovine teeth [33].

**Fig. 2:**
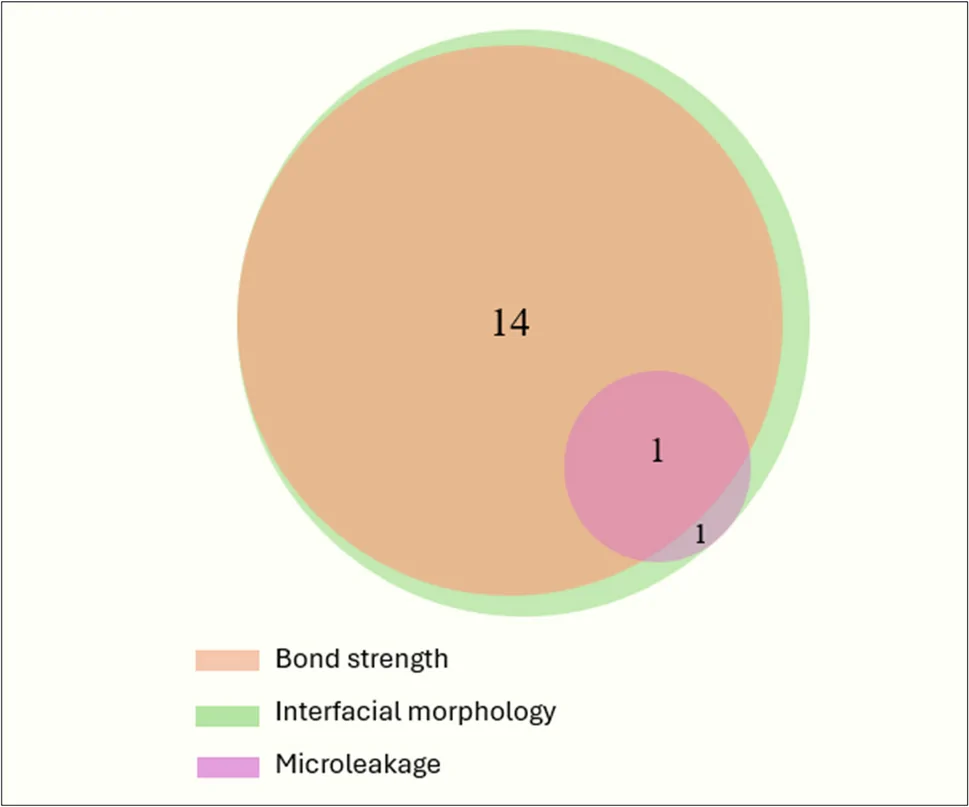
Overlap of outcome parameters assessed across experimental studies included in this scoping review (*n* = 16)

Among the experimental studies, 15 reported quantitative bond-strength data, two assessed microleakage, and all but one provided qualitative interfacial morphology observations. No study has performed a quantitative analysis of remineralization or mineral content changes. The overlap of outcome parameters assessed across the experimental studies is illustrated in Fig. 2.

**Fig. 3:**
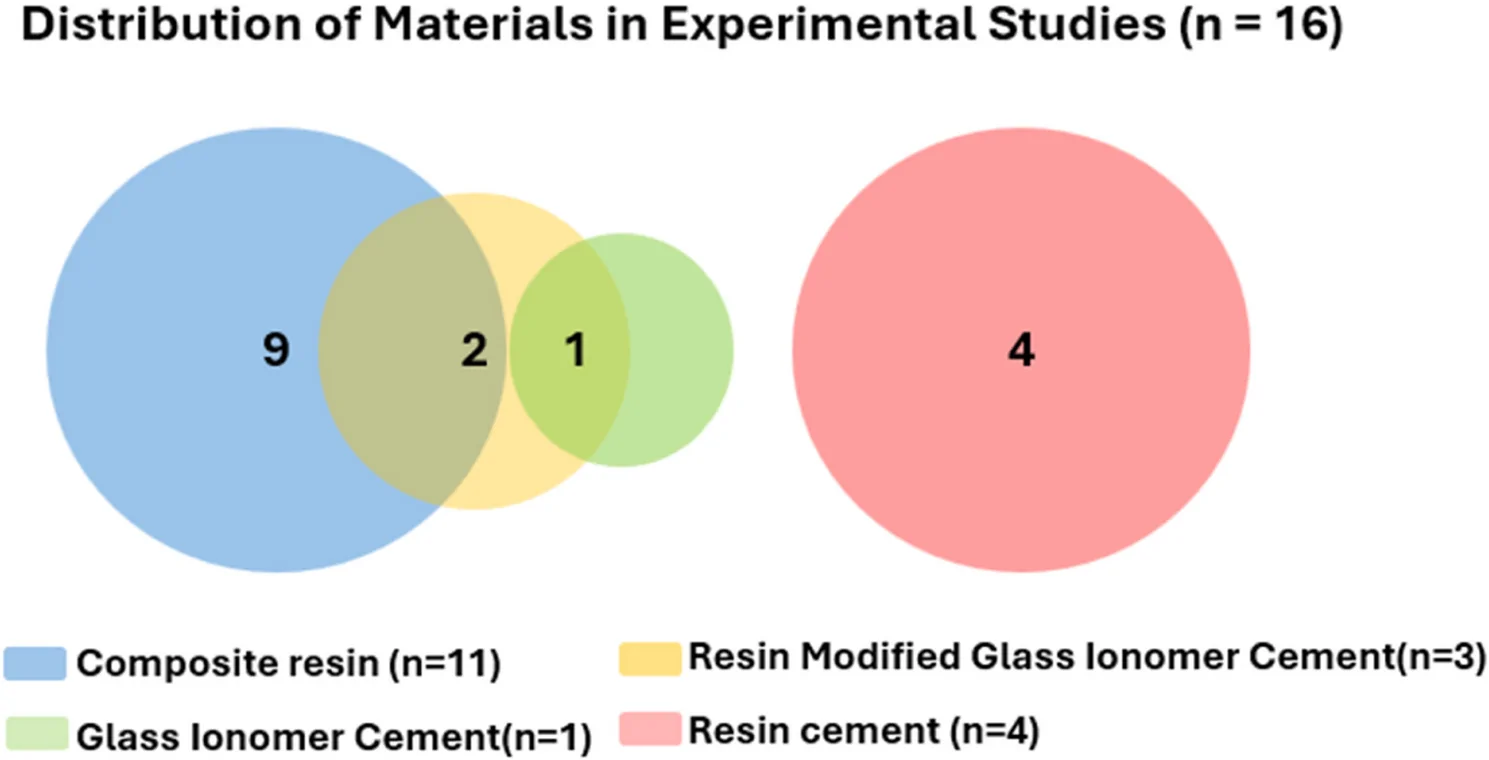
Distribution of materials in experimental studies

Across the experimental studies reporting bond strength outcomes, a range of restorative materials, including composite resins, resin-modified glass-ionomer cements (RMGIC), conventional glass-ionomer cements (GIC), and resin cements, were evaluated. Several studies assessed more than one material. The distribution and overlap of restorative materials evaluated for bond strength testing across experimental studies are illustrated in Fig. 3.

**Fig. 4:**
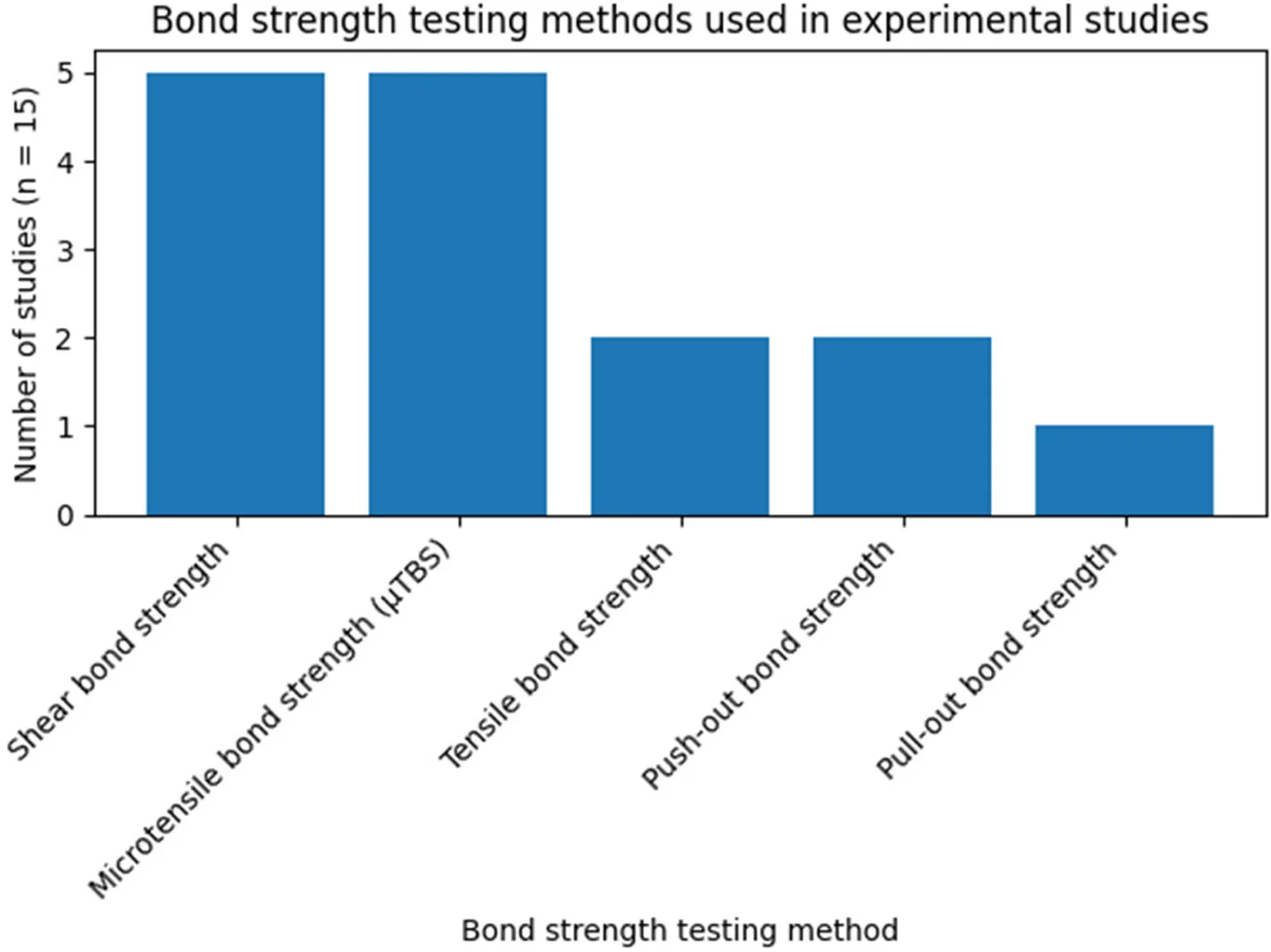
Distribution of bond strength testing methods used in the experimental studies included in this scoping review (*n* = 15)

In experimental studies reporting bond strength outcomes, a range of mechanical testing methods were employed, most commonly shear bond strength and microtensile bond strength (µTBS), followed by tensile, push-out, and pull-out testing (Fig. 4).

The dentin sources were predominantly human permanent premolars or molars. One investigation included bovine teeth in addition to human teeth [33]. The adhesive systems evaluated included universal, etch-and-rinse, and self-etch adhesives, as well as resin-modified glass ionomer cements. AGE accumulation in dentin can be attributed to systemic conditions (e.g., diabetes), natural aging or experimentally induced glycation. The characteristics and key findings of the included studies are presented separately for diabetes-related dentin studies (Table 2 ) and age-related dentin studies (Table 3 ).

**Table 2:** Characteristics and key findings of included diabetes-related dentin studies

Author, year	Study design	Tooth type/source	Dentin substrate/location	Adhesive system/material	Outcome(s) assessed	Key findings
Saghiri et al. 2022 [21]	In vitro experimental study	Extracted caries-free human permanent premolars from non-diabetic, type 1 diabetic, and type 2 diabetic individuals	Flat coronal dentin surfaces	Universal adhesive (3 M Scotchbond/Universal Bonding Agent; 10-MDP-containing adhesive) with restorative composite resin	Tensile bond strength; interfacial morphology	Tensile bond strength was lower in diabetic dentin than in non-diabetic dentin. The type 1 diabetes group showed the lowest tensile bond strength.
Saghiri et al. 2022 [23]	In vitro experimental study	Extracted human permanent molars from non-diabetic, type 1 diabetic, and type 2 diabetic individuals	Flat occlusal enamel and coronal dentin surfaces	Single Bond Universal Adhesive (3 M ESPE; 10-MDP-containing adhesive) with composite resin	Shear bond strength; failure mode/interfacial morphology	Diabetes reduced shear bond strength to enamel and dentin, with the most pronounced reduction observed in the type 1 diabetes group.
Attia et al. 2024 [4]	In vitro experimental study	Extracted sound human molars from non-diabetic, type 1 diabetic, and type 2 diabetic individuals	Mid-coronal dentin surfaces	Scotchbond Universal Adhesive applied in total-etch and self-etch modes with composite resin	Shear bond strength; failure mode/interfacial morphology	Diabetes significantly reduced shear bond strength to dentin. Type 1 diabetes produced the greatest reduction. Self-etch application produced higher bond strength than total-etch application in all groups.
Arslandaş Dinçtürk et al. 2024 [15]	In vitro experimental study	Extracted sound single-rooted human central teeth from diabetic and non-diabetic individuals	Root canal/post-space dentin; middle-third root slices	Variolink N and Panavia SA Universal resin cements, with saline or saline plus 2% chlorhexidine irrigation	Push-out bond strength; failure mode/interfacial morphology	Diabetes reduced bond strength for both resin cements. Chlorhexidine irrigation improved adhesion in diabetic and non-diabetic dentin.

**Table 3:** Characteristics and key findings of included age-related dentin studies

Author, year	Study design	Tooth type/source	Dentin substrate/location	Adhesive system/material	Outcome(s) assessed	Key findings
Sidhu et al. 1991 [24]	In vitro experimental study	Extracted human permanent premolars from younger and older donors	Cervical/Class V cavity dentin at or near the cementoenamel junction	Prisma Universal Bond 2 and Tenure/Marathon dentin adhesive systems	Microleakage; interfacial morphology	Dentin adhesives reduced microleakage compared with controls in both age groups. Tenure/Marathon showed significantly lower microleakage in older dentin, while the other adhesive system showed no clear age-related difference.
Burrow et al. 1994 [29]	In vitro experimental study	Extracted sound human molars from young and older donors	Superficial and deep dentin surfaces prepared from molar dentin discs	Scotchbond Multi-Purpose, Superbond D-Liner, and Liner Bond II	Tensile bond strength; hybrid-layer/interfacial morphology	Dentin depth and age had limited influence on bond strength for most adhesive systems. Deeper dentin tended to show slightly lower bond strength.
Özer et al. 2005 [28]	In vitro experimental study	Extracted sound human mandibular first or second molars from three donor age groups	Flat occlusal enamel and dentin surfaces	Reactmer Bond and ABF Bond	Microtensile bond strength; interfacial morphology	Bond strength was material dependent. ABF Bond showed higher bond strength than Reactmer Bond, and age-related effects differed according to the adhesive system.
Brackett et al. 2008 [27]	In vitro experimental study	Extracted human molars from young adult and older donors	Occlusal mid-coronal dentin and enamel surfaces	Prime & Bond NT and Fuji Bond LC	Microtensile bond strength; SEM/interfacial morphology	No significant age-related reduction in adhesion was observed. SEM evaluation showed differences in etched aged dentin, but aged dentin remained bondable with the tested systems.
Lopes et al. 2011 [32]	In vitro experimental study	Extracted sound human molars from young and older donors	Occlusal dentin surfaces	Adper Single Bond etch-and-rinse adhesive with different acid-etching times	Microtensile bond strength; dentin hardness; interfacial morphology	No correlation was found between microtensile bond strength and dentin hardness. Extending acid-etching time to 30 s improved hybridization and bond strength to old dentin.
Oliveira et al. 2012 [26]	In vitro experimental study	Extracted intact human molars and premolars from three donor age groups	Exposed mid-coronal dentin surfaces	Adper Scotchbond Multi-Purpose Plus, Adper Single Bond Plus, Adper Scotchbond SE, and Adper Easy Bond	Microtensile bond strength; interfacial morphology	Neither chronological age nor etching time significantly influenced dentin bond strength for most tested adhesive systems. Modern adhesives showed minimal age-related effects.
Perdigão et al. 2013 [30]	In vitro laboratory study	Extracted sound human third molars and maxillary premolars from three donor age groups	Middle dentin exposed from crown surfaces	Adper Easy Bond, experimental Adper Easy Bond without Vitrebond copolymer, Adper Single Bond Plus, experimental Adper Single Bond Plus without Vitrebond copolymer, and Adper Scotchbond Multi-Purpose	Microtensile bond strength; interfacial morphology	Dentin age had little effect on bond strength overall. Adhesive composition, particularly the presence of Vitrebond copolymer, influenced bonding performance.
Türk et al. 2017 [25]	In vitro experimental study	Extracted human molars from young and geriatric patients	Buccolingually sectioned coronal enamel and dentin surfaces	Variolink II, Superbond C&B, and Clearfil Esthetic Cement used for glass-ceramic bonding	Shear bond strength; failure mode/interfacial morphology	Aging did not significantly affect glass-ceramic bond strength. Enamel generally contributed more to bond strength than dentin.
Limeira et al. 2019 [33]	In vitro experimental study	Extracted single-rooted human teeth from young and older donors and bovine teeth from young/adult animals	Root canal dentin; coronal, middle, and apical root thirds	RelyX Ultimate with Single Bond Universal and RelyX U200 resin cement	Push-out bond strength; degree of conversion; failure mode/interfacial morphology	Young human root dentin showed higher bond strength than old human root dentin. Aging negatively affected human root dentin but not bovine dentin. Bond strength decreased from coronal to apical root thirds.
Mandra et al. 2020 [31]	In vitro experimental study	Extracted intact human molars from young, elderly, and senile age groups	Coronal crown dentin surfaces	Adper Easy One, Adper Single Bond, and Scotchbond Universal	Microshear bond strength; SEM/interfacial morphology	Age-related changes reduced bond strength to dentin. Universal adhesive showed comparatively stable bonding across age groups.
Techa-Ungkul et al. 2021 [22]	In vitro experimental study	Extracted non-carious human molars from young and aged donors	Middle dentin for microshear testing; cervical cavity dentin/enamel margins for microleakage testing	Fuji II LC, Equia Forte Fil, and Fuji Bulk glass-ionomer cements	Microshear bond strength; microleakage; interfacial morphology	Dentin age did not significantly affect microshear bond strength. Aged dentin showed reduced microleakage, possibly related to tubule occlusion. Resin-modified GIC showed the highest bond strength but greater microleakage than conventional GICs.
Krempels et al. 2025 [11]	In vitro experimental study	Extracted permanent human teeth from young, middle-aged, and older donors	Intracanal/root canal dentin with standardized post-space preparation	Six resin cements, including multistep and self-adhesive systems	Pull-out bond strength; interfacial morphology	Tooth age did not correlate with intracanal dentin adhesion. Resin cement composition was the main determinant of bond strength.

Reporting of participant- and tooth-level variables was variable across the included studies. Most age-related studies reported donor age categories or age ranges, and several studies specified tooth type, dentin location, and storage conditions. In contrast, detailed participant-level clinical information was limited, particularly in studies involving diabetic dentin. HbA1c or glycemic-control status was not reported in the diabetic bonding studies, and medication use and smoking status were not reported in the included primary experimental studies. Diabetes duration was reported in some studies, whereas diabetes type was specified in some but not all diabetic dentin investigations. The degree of sclerosis was generally discussed conceptually or inferred from age-related dentin changes, but it was rarely measured as a specimen-level variable. The evidence base was also methodologically heterogeneous. The included studies differed in dentin condition, tooth type, dentin location, adhesive system, restorative material, storage protocol, and bond-strength test method. Substrates included diabetic and non-diabetic dentin, young and aged dentin, coronal dentin, cervical dentin, intracanal root dentin, root thirds, and, in one study, bovine dentin. Therefore, the results were synthesized descriptively rather than pooled quantitatively.

### Bond strength outcomes

The included studies collectively evaluated the influence of systemic disease and aging on resin dentin adhesion.

### Diabetic dentin

Two related studies by Saghiri and coworkers revealed that diabetes mellitus significantly reduced bond strength: the tensile strength to diabetic dentin was markedly lower than that to healthy dentin, with type 1 diabetes causing the greatest reduction [21], and the shear bond strength to enamel and dentin was similarly diminished in diabetic groups [23]. Attia et al. reported that universal adhesive performance was impaired on diabetic dentin, although the self-etch mode produced slightly higher bond strength than the total-etch mode did, and type 1 diabetes remained the most detrimental [4]. Arslandaş Dinçtürk et al. reported that chlorhexidine irrigation improved bond strength in both diabetic and non-diabetic dentin and that a total-etch resin cement outperformed a self-adhesive cement in diabetic teeth [15].

### Age-related dentin

Many studies have compared the adhesion of various bonding systems to young versus aged dentin. Sidhu et al. reported that adhesives significantly reduced microleakage, but older dentin exhibited greater leakage, and glycidyl-methacrylate adhesives performed better than HEMA-based systems did [24]. Burrow et al. (1994) reported that the bond strength decreased with increasing dentin depth but was relatively unaffected by donor age [29]. Subsequent investigations using contemporary adhesives generally reported minimal age-related differences in bond strength. Ӧzer et al. (2005) reported that age decreased bond strength for a one-step glass-ionomer-based adhesive but increased strength for a two-step antibacterial adhesive [28]; Brackett et al. (2008) reported no significant differences in microtensile bond strength between young and aged teeth [27]; Oliveira et al. concluded that neither chronological age nor etching time markedly influenced bond strength [26]; and Perdigao et al. (2013) reported that inclusion of a Vitrebond copolymer improved bonding regardless of dentin age [30]. An extended etching time restored the bond strength to levels comparable to those of young dentin [32]. Several later studies corroborated these findings: Turk et al. reported no significant agerelated reduction in bond strength to lithium-disilicate ceramics [25], and Krempels et al. reported that tooth age did not affect intracanal pull-out bond strength, with the type of cement being the main determinant [11]. Nevertheless, Limeira et al. reported that aging reduced push-out bond strength in human root dentin, although bovine dentin was unaffected [33]. Mandra et al. reported that hypermineralized, sclerotic dentin reduced bond strength across adhesives, whereas universal adhesives offered the most stable adhesion [31].

### Glass-ionomer and resin-cement systems

Techa-Ungkul and Sakoolnamarka evaluated three glass-ionomer cements on young and aged molars and reported that dentin age did not influence bond strength, although aged dentin exhibited reduced microleakage; resin-modified glass ionomer (Fuji II LC) achieved the highest bond strength overall [22]. Turk et al. studied lithium disilicate ceramics cemented to young and geriatric dentin with two etch-and-rinse and one self-etch resin cement and reported no significant age effect; etch-and-rinse systems remained preferable across age groups [25]. Arslandaş Dinçtürk et al. compared a total-etch dual-cure cement (Variolink N) with a self-adhesive universal cement (Panavia SA Universal) and reported that chlorhexidine irrigation improved bond strength in both diabetic and non-diabetic dentin; in diabetic teeth, the total-etch cement outperformed the self-adhesive cement [15]. Limeira et al. measured the push-out bond strength of two resin cements to human and bovine root dentin; aging significantly reduced the bond strength in human root dentin but not in bovine samples, and values decreased from the coronal to the apical thirds [33].

### Interfacial morphology and microleakage

Several investigations have included microscopic analyses and microleakage assessments. Sidhu et al. reported that the Tenure/Marathon dentin adhesive system, which contains N-(p-tolyl) glycine glycidyl methacrylate (NTG-GMA), produced significantly less microleakage at the tooth-restoration interface in teeth from patients over 55 years of age than in those from patients under 20 years of age [24]. Ӧzer et al. reported that SEM evaluation of the resin-dentin interface revealed a thin hybrid layer for both adhesive systems, with perfect marginal adaptation and no interfacial gaps observed after argon-ion beam etching [28]. Techa-Ungkul and Sakoolnamarka reported less microleakage in aged dentin, possibly due to increased tubule occlusion [22]. These morphological observations support the notion that tubule occlusion and adhesive strategies influence the quality of the hybrid layer and sealing ability. No included study attempted a quantitative meta-analysis because of heterogeneity in substrates, adhesives, and testing protocols.

### Remineralization and mineral content outcomes

None of the included studies performed quantitative remineralization or mineral content analyses. One experimental study discussed fluoride release from glass-ionomer-based adhesive materials as a potential contributor to substrate interaction, but remineralization was not directly measured [28].

## Discussion

### Summary of evidence

This scoping review identified 16 primary experimental studies evaluating adhesive performance on dentin affected by aging, diabetes, or related substrate modifications, with most evidence derived from in vitro experiments on extracted teeth. Fifteen investigations reported quantitative bond strength measurements, and two assessed microleakage; quantitative remineralization analyses were not reported. The evidence base was highly heterogeneous with respect to dentin condition, species, tooth type, dentin location, adhesive system, restorative material, storage protocol, and bond-strength test method. Therefore, the findings should be interpreted as a descriptive map of available evidence rather than as pooled evidence supporting a single effect estimate. Only four included studies directly evaluated diabetic dentin; these studies generally reported lower bond strength than non-diabetic dentin, particularly in type 1 diabetes. In contrast, most included studies evaluated age-related dentin, where responses were more heterogeneous: several studies using contemporary adhesives reported little difference between young and aged dentin, whereas others reported reduced push-out strength, altered interfacial morphology, or differences in microleakage. Importantly, none of the included bonding studies directly quantified AGE levels in dentin before adhesive testing. Therefore, although AGE accumulation is biologically plausible in aging and diabetes, the available evidence does not permit direct causal attribution of bonding outcomes to AGE accumulation. Across the included in vitro studies, some universal adhesives (often 10-MDP-containing adhesives) and RMGICs showed comparable or higher immediate bond strengths in certain protocols, but the results varied by substrate region, testing method, and application mode.

### Conceptual distinction between aging, diabetes, and AGE accumulation

A central issue emerging from this review is the need to distinguish between aging, diabetes, and AGE accumulation. Aging is associated with dentinal sclerosis, tubule occlusion, reduced permeability, and gradual collagen and mineral changes. Diabetes, however, represents a systemic metabolic condition in which hyperglycemia, oxidative stress, inflammation, altered mineral metabolism, and disease-related variables such as duration and glycemic control may influence dentin [34]. AGE accumulation may contribute to substrate alteration in both contexts, but it is not the only mechanism and was not directly measured in the bonding studies included in this review. Therefore, the terms aged dentin, diabetic dentin, and glycated dentin should not be used interchangeably. The evidence is best interpreted as showing that aging and diabetes are dentin-modifying conditions that may affect bonding, while the specific role of AGEs remains an important but unresolved mechanistic question.

### Potential biological mechanisms underlying substrate-related bonding differences

AGE formation is a non-enzymatic process promoted by hyperglycemia and aging; reducing sugars react with collagen to produce cross-linked matrices that stiffen the tissue and alter its mechanical properties [35, 36]. A recent systematic review revealed that diabetes and AGE accumulation negatively influence the physical and mechanical properties of dentin, although the evidence remains limited [37]. Laboratory studies have shown that naturally aged dentin contains higher AGE levels and that increased AGE content is correlated with reduced flexural strength and toughness [8]. Ribose-induced glycation experiments demonstrated that glycation significantly increased the elastic modulus and ultimate tensile strength of dentin while not affecting the energy to fracture, indicating that glycated dentin becomes stiffer but not necessarily more resilient [35, 38]. In diabetic animal models and human specimens, the microhardness is significantly reduced, and the dentinal tubule density and concentrations of trace elements (e.g., magnesium and strontium) are decreased, suggesting that systemic disease affects both organic and inorganic components of dentin [39]. These structural alterations may reduce permeability, stiffen collagen, and alter mineral composition, potentially impeding adhesive monomer diffusion and hybrid-layer formation [26, 28, 39, 40]. However, their contribution to bonding outcomes could not be directly confirmed because the included adhesive studies did not quantify AGE levels. Furthermore, hyperglycemia induces oxidative stress and upregulates matrix metalloproteinases, potentially accelerating collagen degradation within the hybrid layer [4, 41–43].

### Influence of the adhesion strategy and material on the bond strength

Across the heterogeneous included studies, adhesive performance appeared to depend strongly on material composition and application strategy. Some studies suggested advantages for universal adhesives incorporating functional monomers, particularly 10-MDP, but the evidence was not uniform across dentin conditions, test methods, and substrate locations. Saghiri and coworkers reported that diabetes significantly reduced tensile and shear bond strength [21, 23]. However, compared with alternative adhesives, 10-MDP-containing universal adhesive (Scotchbond Universal; 3M ESPE) consistently yielded higher values, and type 1 diabetes was associated with the greatest reduction. Similarly, Attia et al. reported that the bond strength of a 10-MDP-based universal adhesive (Scotchbond Universal; 3M ESPE) decreased in both type 1 and type 2 diabetic dentin, with type 1 samples showing the lowest shear bond strength; importantly, applying the adhesive in self-etch mode resulted in higher bond strength than the total-etch mode did in all groups [4]. In a push-out test on fiber posts, chlorhexidine irrigation significantly improved bond strength in both diabetic and non-diabetic dentin, whereas total-etch, dual-cure resin cement (Variolink N; Ivoclar Vivadent) resin cement outperformed self-adhesive resin cement (Panavia SA Universal; Kuraray), indicating that MMP inhibition and resin chemistry influence adhesion [15]. These findings suggest that chemical bonding via functional monomers and enzymatic inhibition by chlorhexidine may improve bonding in compromised dentin substrates; however, the included studies did not directly determine whether these effects were related to AGE accumulation.

Age-related dentin responses depend on the adhesive composition. Early work by Sidhu et al. revealed greater microleakage in older cervical dentin and reported that a NTG-GMA-containing adhesive (Tenure/Marathon) reduced leakage more effectively than a HEMA/glutaraldehyde system did [24]. Burrow et al. reported that donor age had little influence on tensile bond strength when modern etch-and-rinse adhesives were used, whereas dentin depth was more important [29]. Perdigao et al. demonstrated that adding a Vitrebond copolymer to a one-step self-etch adhesive improved bond strength regardless of dentin age [30]. Krempels et al. reported no correlation between age and pull-out bond strength when six types of resin cements were compared; instead, the cement composition determined the performance [11]. Limeira et al. reported that aging reduced the push-out bond strength to human root dentin and that the bond strength decreased from the coronal to the apical thirds of the root [33]. Mandra et al. reported that hypermineralized sclerotic dentin lowered the bond strength across adhesives, but universal adhesives provided the most stable adhesion [31]. In glass-ionomer systems, Techa-Ungkul and Sakoolnamarka reported that compared with conventional glass-ionomer cement, resin-modified glass-ionomer cement achieved higher bond strength across young and aged dentin and that aged dentin exhibited less microleakage [22]. Taken together, these data suggest that adhesives containing 10-MDP or other functional monomers and resin-modified glass-ionomer cements offer robust bonding across age groups and that application mode matters, particularly in diabetic dentin.

### Microleakage, interfacial morphology and remineralization

Only two included studies evaluated microleakage. Sidhu et al. (1991) reported that both dentin adhesives tested reduced microleakage compared with controls, with lower microleakage reported in teeth from patients over 55 years of age when an NTG-GMA containing adhesive was used [24]. In contrast, Techa-Ungkul and Sakoolnamarka reported reduced microleakage in aged dentin for both resin-modified and conventional glass-ionomer cements; however, resin-modified glass-ionomer cement exhibited higher leakage overall [22]. Differences in microleakage outcomes between these studies may partly reflect methodological variations, including cavity design and substrate location (root/cervical dentin versus standardized cervical cavities with defined enamel and dentin margins), restorative materials evaluated (early-generation resin adhesives versus glass-ionomer cements), and dye penetration protocols and scoring systems. In particular, the chemical bonding mechanism and fluoride release of glass-ionomer cements, together with increased tubule occlusion in aged dentin, may have contributed to the reduced leakage observed in the latter study.

Previous studies have reported thinner hybrid layers, shorter resin tags and more interfacial defects in diabetic or aged dentin than in healthy dentin [15, 30]. Nevertheless, the morphological descriptions were qualitative and inconsistent across the studies. No included study quantified remineralization or mineral content. Although fluoride release from glass-ionomer-based materials may theoretically support remineralization, the included experimental studies did not directly measure this outcome. Therefore, the effects of AGE accumulation on remineralization remain unclear.

### Supporting evidence on AGE-related mechanical changes in dentin

Microhardness tests revealed that type 1 diabetes significantly reduced dentin microhardness and tubule density and decreased the levels of trace elements such as magnesium, strontium and zinc. The mineral density and thickness of enamel and dentin were reduced in diabetic rats, reflecting hypoplastic changes [39]. Studies employing nanoindentation reported that compared with control samples, ribose-treated dentin samples had a significantly higher elastic modulus, indicating that glycation stiffens dentin matrices [38]. Comparisons of flexural strength and toughness across age groups revealed a negative correlation between AGE accumulation and mechanical strength; aged dentin presented greater mineral density and increased tubule occlusion but lower toughness and flexural strength [8]. Alania et al. reported that ribose-induced glycation significantly increased the ultimate tensile strength and elastic modulus but did not affect the energy to fracture, suggesting that glycated dentin becomes brittle rather than resilient [35]. Collectively, these supporting mechanistic studies indicate that AGE accumulation can alter the mechanical properties of dentin. However, because the included adhesive studies did not directly quantify AGE levels before bonding, AGE-related mechanical alteration should be interpreted as a plausible explanatory pathway rather than a demonstrated cause of the bonding outcomes observed in this review.

### Clinical implications

Diabetes and population aging mean that clinicians will increasingly encounter dentin substrates affected by age-related sclerosis, altered permeability, systemic metabolic disease, and potentially increased collagen glycation. The evidence from this review suggests that compared with age-related dentin, diabetic dentin has a more pronounced reduction in bond strength, particularly when type 1 diabetes is present. Universal adhesives containing functional monomers such as 10-MDP appear to offer the most reliable bonding across diabetic and aged substrates, especially when applied in self-etch mode, and chlorhexidine irrigation can further increase adhesion by inhibiting matrix metalloproteinases [15]. Clinicians should follow the manufacturers’ guidance for active application and thorough solvent evaporation and may consider immediate dentin sealing for indirect restorations, as this technique improves bond strength and durability [44]. Compared with conventional resin adhesives, RMGIC may be advantageous in geriatric patients, as the chemical bonding mechanism of RMGIC appears to be less affected by age-related dentin changes, and their clinical handling is generally considered less technique sensitive [22, 27, 45]. Because no included study quantified remineralization or directly measured AGE levels in dentin before adhesive testing, there is insufficient evidence to recommend remineralizing or adhesive strategies specifically for AGE-mediated dentin changes. Clinical interpretation should therefore be based on the broader substrate context, including diabetes status, donor age, dentin location, degree of sclerosis, and the adhesive strategy used.

### Limitations and research gaps

The main conceptual limitation of this review is that aging and diabetes were used as clinical or biological contexts in which dentin substrate changes may occur, but the included bonding studies did not directly measure AGE accumulation. Therefore, the review cannot determine whether reduced bond strength or altered interfacial morphology was caused by AGEs, dentinal sclerosis, altered mineral content, reduced tubule density, diabetes-related metabolic changes, or other participant-level factors.

Another important limitation is the imbalance in the evidence base. Most included studies investigated age-related dentin, whereas only four directly evaluated diabetic dentin. Therefore, conclusions regarding diabetic dentin should be interpreted cautiously and should be considered less robust than conclusions regarding age-related dentin. In addition, the included studies varied substantially in dentin source, tooth type, substrate location, adhesive system, storage method, aging protocol, and mechanical testing approach. This heterogeneity precluded quantitative pooling and limits the extent to which broad clinical conclusions can be drawn.

This review is limited by the predominance of in vitro studies, heterogeneous dentin sources, inconsistent adhesive protocols and outcome measures. These reporting gaps limited interpretation of the evidence. Tooth-level variables also differed across studies, including tooth type, coronal versus root dentin, cervical dentin, intracanal root dentin, and root thirds. Storage protocols also varied, including chloramine-T, thymol, saline, distilled water, freezing, and humid incubation. These differences may influence dentin hydration, permeability, collagen condition, and adhesive performance, but their effects could not be formally separated from those of aging or diabetes. This limitation is particularly relevant for diabetic dentin, because a young patient with well-controlled diabetes and an older patient with long-standing poorly controlled diabetes may present substantially different dentin substrate characteristics. Future research should therefore incorporate direct AGE quantification, such as immunostaining or spectroscopic assays, alongside bond-strength measurements to clarify the relationship between AGE content and adhesion. Long-term in situ and clinical studies comparing adhesives on diabetic and aged dentin are needed, with standardized protocols, thermocycling or pH cycling, and quantitative assessments of microleakage, remineralization and mechanical properties. Investigations into new cross-linking agents, antioxidants, bioactive fillers, and pretreatments could help identify adhesive strategies better suited for aged, diabetic, or experimentally glycated dentin substrates.

## Conclusion

Dentin affected by diabetes or aging may present altered adhesive performance, but the available evidence is heterogeneous, mainly derived from in vitro studies, and weighted more heavily toward age-related dentin than diabetic dentin. In the limited number of diabetes-related studies, diabetic dentin generally showed lower bond strength than non-diabetic dentin, whereas findings for age-related dentin were more variable and appeared to depend on adhesive composition, dentin location, and testing method. Although AGE accumulation is a biologically plausible contributor to dentin substrate alteration, none of the included bonding studies directly quantified AGE levels; therefore, the observed bonding outcomes cannot be attributed specifically to AGEs. Future studies should separately evaluate aged, diabetic, and experimentally glycated dentin, quantify AGE accumulation or related biochemical markers, and report clinically relevant confounders including donor age, diabetes type, diabetes duration, HbA1c or glycemic-control status, medications, smoking, comorbidities, tooth type, dentin location, degree of sclerosis, storage medium, and storage duration.

## Supplementary Information

Supplementary Material 1.

Supplementary Material 2.

## Data Availability

The datasets used and/or analyzed in the present study are available from the corresponding author upon reasonable request.

## Abbreviations

AGEs: Advanced glycation end products
Bis-GMA: Bisphenol A glycidyl methacrylate
CEL: Carboxyethyllysine
CML: Carboxymethyllysine
F-PRG: Fluoro-alumino-silicate pre-reacted glass filler
GIC: Glass-ionomer cement
HEMA: 2-Hydroxyethyl methacrylate
JBI: Joanna Briggs Institute
MMA: Methyl methacrylate
MMPs: Matrix metalloproteinases
NTG-GMA: N-(p-tolyl) glycine glycidyl methacrylate
OSF: Open Science Framework
PCC: Population-Concept-Context
PRISMA-ScR: Preferred Reporting Items for Systematic Reviews and Meta-Analyses Extension for Scoping Reviews
RMGIC: Resin-modified glass-ionomer cement
SEM: Scanning electron microscopy
TEGDMA: Triethylene glycol dimethacrylate
µTBS: Microtensile bond strength

## Declarations

Not applicable, as this scoping review involved analysis of previously published literature and did not involve human participants or identifiable patient data.
